# Advocacy for the Medicinal Plant *Artabotrys hexapetalus* (Yingzhao) and Antimalarial Yingzhaosu Endoperoxides

**DOI:** 10.3390/molecules27196192

**Published:** 2022-09-21

**Authors:** Christian Bailly, Jean-Pierre Hénichart

**Affiliations:** 1 OncoWitan, Scientific Consulting Office, 59290 Lille (Wasquehal), France; 2 Institut de Chimie Pharmaceutique Albert Lespagnol (ICPAL), Faculty of Pharmacy, University of Lille, 3 Rue du Professeur Laguesse, 59000 Lille, France

**Keywords:** *Artabotrys hexapetalus*, endoperoxide, malaria, natural products, yingzhaosu

## Abstract

The medicinal plant *Artabotrys hexapetalus* (synonyms: *A.* *uncinatus* and *A. *odoratissimus**) is known as yingzhao in Chinese. Extracts of the plant have long been used in Asian folk medicine to treat various symptoms and diseases, including fevers, microbial infections, ulcers, hepatic disorders and other health problems. In particular, extracts from the roots and fruits of the plant are used for treating malaria. Numerous bioactive natural products have been isolated from the plant, mainly aporphine (artabonatines, artacinatine) and benzylisoquinoline (hexapetalines) alkaloids, terpenoids (artaboterpenoids), flavonoids (artabotrysides), butanolides (uncinine, artapetalins) and a small series of endoperoxides known as yingzhaosu A-to-D. These natural products confer antioxidant, anti-inflammatory and antiproliferative properties to the plant extracts. The lead compound yingzhaosu A displays marked activities against the malaria parasites *Plasmodium falciparum* and *P. berghei*. Total syntheses have been developed to access yingzhaosu compounds and analogues, such as the potent compound C14-epi-yingzhaosu A and simpler molecules with a dioxane unit. The mechanism of action of yingzhaosu A points to an iron(II)-induced degradation leading to the formation of two alkylating species, an unsaturated ketone and a cyclohexyl radical, which can then react with vital parasitic proteins. A bioreductive activation of yingzhaosu A endoperoxide can also occur with the heme iron complex. The mechanism of action of yingzhaosu endoperoxides is discussed, to promote further chemical and pharmacological studies of these neglected, but highly interesting bioactive compounds. Yingzhaosu A/C represent useful templates for designing novel antimalarial drugs.

## 1. Introduction

Despite new treatment modalities, malaria remains a major public health threat worldwide, causing more than 400,000 deaths per year, predominantly in children in sub-Saharan Africa and tropical regions [1]. Recently, the malaria vaccine RTS,S (Mosquirix™) has received regulatory approval for the prevention of malaria infection. The launch of this vaccine represents a major achievement in limiting the transmission of the parasite, but it shows a modest efficacy against malaria illness [2]. New treatments are still needed to combat the disease, notably the drug-resistant forms, which develop rapidly. There is a significant need for efficient, affordable and well-tolerated antimalarial drugs [3].

The first-line treatment of *Plasmodium falciparum* mild malaria generally relies on combination therapy, including artemisinin (ART) and/or chloroquine, whereas intravenous artesunate is often preferred in cases of severe malaria, at least in developed countries [4]. The combination of artesunate and pyronaridine (Pyramax^®^) is also approved to treat uncomplicated malaria [5,6]. ART and derivatives have been extensively studied as antimalarial drugs. The discovery of artemisinin was largely based on traditional Chinese medicine (TCM), as recognized by Dr. Youyou Tu, who was awarded the Nobel Prize in Physiology and Medicine in 2015 for her discovery of ART and its therapeutic effects on malaria [7]. Herbs of the *Artemisia* family (qinghao in Chinese) have been extensively studied for their pharmacological properties and phytochemical content.

**Figure 1:**
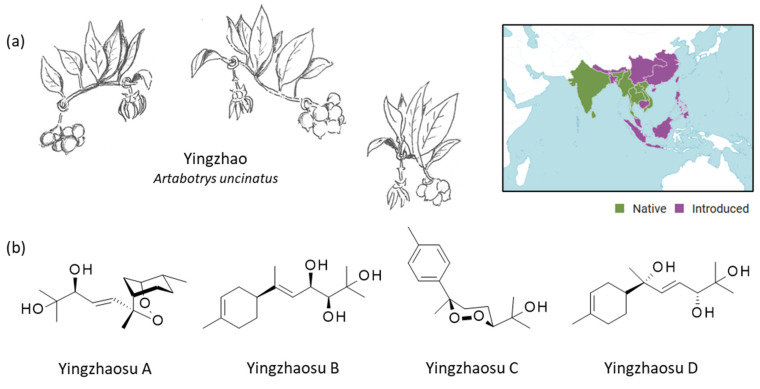
Yingzhao plant and yingzhaosu compounds. (**a**) The plant yingzhao (*Artabotrys uncinatus* (L.) Meer.) with a view of the young fruits, the leaves and the flower (drawing: Prof. J.-P. Hénichart). The plant is largely distributed in Southeast Asia. The plant is native to countries such as India, Thailand, Vietnam, and Sri Lanka (and other counties in green) and has been introduced in Indonesoia, China, Japan (and other countries in purple) (https://powo.science.kew.org/taxon/urn:lsid:ipni.org:names:72395-1, accessed on 14 September 2022). (**b**) Structure of the four yingzhaosu compounds, all isolated from yingzhao.

In contrast, the plant yingzhao (*Artabotrys unciatus* (L.) Meer.) has been considerably less investigated. Moreover, the main phytochemical compounds found in yingzhao, known as yingzhaosu, have been essentially neglected, at least from a pharmacological viewpoint (Figure 1). The present review offers a survey of the current knowledge and recent research about this plant and its bioactive constituents.

Specifically, the review presents the origin and use of the plant yingzhao, with a detailed analysis of the phytochemicals isolated from the different parts of the plant. Their mechanisms of action are discussed, focusing on the yingzhaosu products to highlight the reactivity of the endoperoxide compounds yingzhaosu A and C. The significance of the work reported here is high considering the need for new drugs to treat malaria but also a novel targeted approach to treating other parasitic diseases and cancers. Artemisinin-type drugs are increasingly considered to treat hematological malignancies, viral infections and other human diseases [8,9,10]. The importance of the reactive endoperoxide function in chemistry is well recognized, and the use of naturally occurring endoperoxides to design new drugs has often been underlined [11,12]. In this context, it is timely and essential to promote yingzhaosu compounds.

## 2. The Plant Yingzhao

The Chinese medicine yingzhao is usually associated with the plant name *Artabotrys uncinatus* (L.) Meer. (Annonaceae), from which yingzhaosu compounds have been isolated, as discussed above (Figure 1). However, and in fact, *A. unciatus* is one of the synonyms for the plant *Artabotrys hexapetalus* (L.f.) Bhandari, which is the accepted botanical name [13]. Other synonyms are used, such as *A. odoratissimus* R.Brown. (Table 1). This plant, commonly called tail grape or climbing ylang-ylang, can be found in China, India, Malaysia, Indonesia, Vietnam and other countries of Southeast Asia. It is native to Sri Lanka and Southern India and can be grown in many places [14]. For example, two established plants of *A. hexapetalus* (L.f.) Bhand. growing at the Fairchild Tropical Garden in Miami (US) have been used to study plant architecture during growth [15]. It can also be found in some tropical African countries, like Tanzania [16].

Yingzhao behaves as a woody climber, possibly growing up to 10 m in height. It should not be confused with *Cananga odorata* (ylang-ylang) used in perfumery. *A. hexapetalus* is a woody scandent climbing shrub. The leaves are oblong to broadly lanceolate in shape and the flowers are fragrant, with yellow petals (Figure 1). The plant is recognizable from the flower stalks, which are shaped like hooks. With beautiful and aromatic flowers, it is an ornamental plant now cultivated and also used in the perfume industry. The seeds are used for propagation, but the germination process is complex and long, taking as long as 238 days [17]. The plant is appreciated for its pleasant-smelling yellow flowers, from which a delicate essential oil, rich in sesquiterpenoids (such as β-caryophyllene and caryophyllene oxide), can be prepared [18]. Essential oils can also be obtained from the stem bark or leaves of the plant to be used as a mosquito repellent [16].

## 3. Traditional Medicinal Uses of Yingzhao

For a long time, yingzhao-based preparations have been used to treat human diseases. Different parts of the plant have been used. The symptoms and pathologies treated with plant extracts vary from one country to another. In Malaysia, leaf decoctions were given for curing cholera, whereas the roots and fruits have been used for the treatment of malaria and scrofula (tuberculous lymphadenitis). There is also mention of the use of the plant to treat fever, diarrhea, dysentery, cuts, sprains, ulcers, and asthma.

Recently, Kousalya and Doss [19] have inventoried the diverse ethnomedicinal uses of *A. hexapetalus* extracts. These can range from antibacterial and antifungal effects to hepato-protective and anti-ulcer activities (Table 2). In general, the pharmacological effects were obtained with organic extracts prepared from the plant’s leaves, bark, or roots. For example, methanolic leave extracts were found to display antifungal and antibacterial effects [20,21,22]. The essential oil of *A. odoratissimus* R.Br. (synonym) has shown a broad-spectrum activity against 14 different storage fungi, and interestingly, it was reported to arrest aflatoxin B1 secretion by a toxic strain of *Aspergillus flavus* [23]. Flower extracts of *A. hexapetalus* also revealed antifungal effects [24].

Alcoholic leave extracts display cytoprotective effects in vitro and in vivo. An ethanolic extract orally administered (100–200 mg/k for seven days) to mice with drug-induced liver injury was found to reduce the oxidative damages at least at the biochemical level and to alleviate the sign of cellular degeneration and necrosis. The extract was well tolerated in mice [40]. The observed effects were attributed to the presence of antioxidant natural products, including flavonoids and alkaloids. Similar antioxidant and hepatoprotective effects have been reported in other studies with *A. hexapetalus* extracts [29,37,38].

The hydroalcoholic leave extracts of *A. hexapetalus* have been found to reduce sperm count and mobility in rats, reducing the diameter of seminiferous tubules. The extract lowered the testosterone level and significantly reduced fertility in rats [33]. The observation was consistent with the reported antifertility activity of various leaf extracts of *A. odoratissimus* Roxb. (synonym). In this case, the extracts (obtained with benzene, ethanol and water) were found to disturb the oestrus cycle and reduce implantation and thus fertility [31]. Antifertility activity has been confirmed in a recent study performed with extracts from the leaves and stem of *A. odoratissimus* Roxb. in female rats. The extracts altered the level of cholesterol and steroidal hormones (estradiol and progesterone) and caused polycystic ovaries in rats [33,34]. In India, antifertility activity of *A. odoratissimus* plant extracts is known for several decades [41,42] and remains considered today for the regulation of fertility [35].

**Figure 2:**
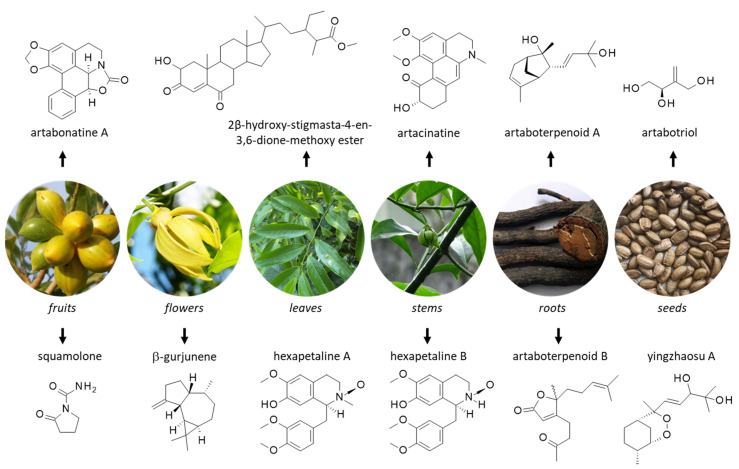
Structure of selected natural products isolated from different parts of yingzhao. Detailed botanical information on yingzhao can be found at http://www.instituteofayurveda.org/plants/plants_detail.php?i=75&s=Local_name (accessed on 14 September 2022).

The main bioactivity of *A. hexapetalus* refers to antiparasitic effects. For a long time, yingzhao (roots and fruits) has been used to combat parasites such as *Plasmodium falciparum* and *Leishmania donovani*. A petroleum ether extract of *A. hexapetalus* was shown to moderately reduce the growth of the promastigote forms of cultured *L. donovani*. The effect was attributed to the presence of flavonoids such as quercetin and apigenin [28]. Notwithstanding, there are many other flavonoids in the plant extracts, such as taxifolin, apigenin glycosides, glucoluteolin, arabotrysides A and B (Figure 2), and the flavonol glycosides called arapetalosides A and B [43,44]. Hydroalcoholic leaf extracts of *A. hexapetalus* display activities against *Plasmodium* and *Leishmania*, but not against the African earthworm *Eudrilus eugeniae* (African nightcrawler) [27,28]. It should be noted that there is not many published information about the antiplasmodial activity of *A. hexapetalus*. Solid data have been reported with other species, such as *A. crassifolius* [45], but not for yingzhao extracts despite the traditional use.

**Figure 3:**
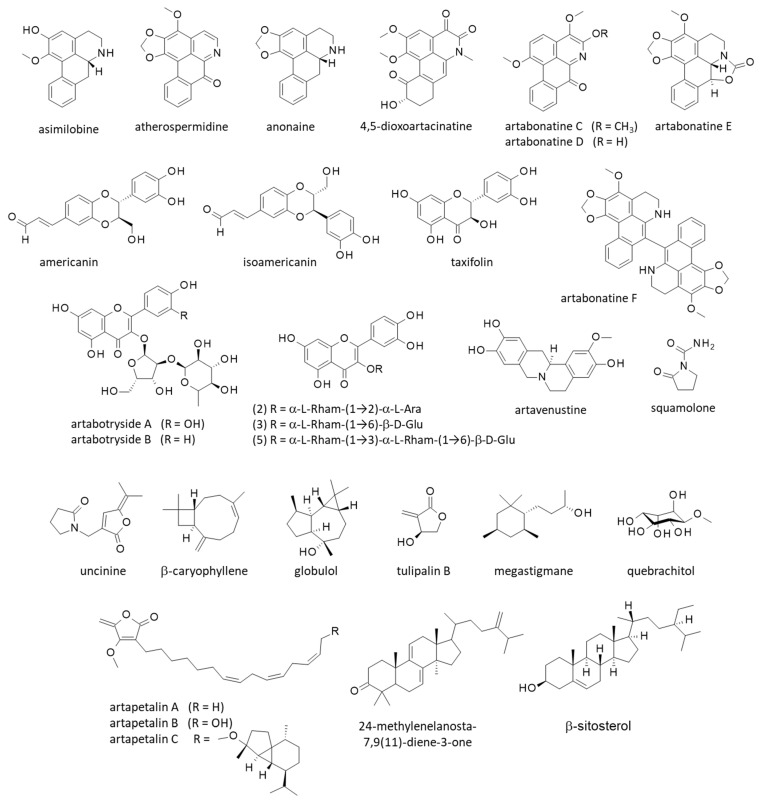
Structure of other natural products isolated from yingzhao.

Occasionally, other pharmacological effects have been reported. For example, a recent study underlined the antiproliferative activity of *A. odoratissimus* fruit extract against MIA PaCa-2 pancreatic cancer cells. The organic (ethyl acetate) extract reduced cell growth, revealed an antioxidant effect, and induced apoptotic cell death associated with DNA damage [36]. The phytochemicals at the origin of the anticancer action were not specified, but it could be linked to the presence of cytotoxic alkaloids. More than 25 alkaloids have been isolated from *A. uncinatus* (synonym), including cytotoxic oxoaporphines and other alkaloids endowed with cytotoxic effects such as atherospermidine and squamolone (Figure 3) [46,47].

The multiple bioactive properties evidenced by extracts of the plants stimulate research and the elaboration of novel bioinspired products. For example, silver nanoparticles have been made using an aqueous extract of *A. hexapetalus* with the objective to propose new bactericide products [30]. There are also non-medicinal usages of the plant extracts. For example, the plant leaf extract has shown anticorrosion activity. It could be used as an eco-friendly green inhibitor for acidic-induced corrosion of mild steel [48,49].

## 4. Phytochemical Content of Yingzhao

Unsurprisingly, a large number of secondary metabolites have been isolated from yingzhao, including terpenoids, alkaloids and steroids. Natural products have been identified from extracts of all parts of the plant, from roots to leaves, and from seeds to fruits. Important products identified from each part are indicated in Figure 2 and Figure 3.

### 4.1. Alkaloids from Yingzhao

The oxazoloaporphine alkaloids artabonatines A-F have been isolated from fresh unripe fruits of *A. uncinatus* [46,47]. They are rare compounds, scarcely studied apart from the chemical synthesis of some derivatives [50]. The structure (from *anti* to *syn*) of artabonatine A has been revised in 2018, based on the chemical synthesis of the two diastereomeric isomers of (−)-artabonatine A (Figure 2). Their capacity to inhibit G-protein coupled receptors (GPCR) and monoamine transporters were characterized. The *anti*-isomer was found to function as a potent inhibitor of serotonergic 5-HT_2C_ receptor (K_i_ = 1.6 μM) whereas the *syn*-isomer inhibited dopamine transporter (K_i_ = 3.8 μM) [50]. These compounds have been rarely identified in other plants. Artabonatine B has been found in the stems of *Annona cherimola* Mill (cherimoya), which is also a tropical species of Annonaceae [51]. Another aporphine alkaloid, 8-hydroxyartabonatine C, has been found in the leaves and twigs of *Pseuduvaria trimera* and the compound was shown to exhibit mild cytotoxic properties toward cancer cells in vitro [51]. However, apart from those two studies, the artabonatine compounds are poorly known. Two related benzylisoquinoline alkaloids named hexapetalines A and B (Figure 2) have been isolated from the stem of *A. hexapetalus*. Hex-A proved to be more cytotoxic toward cancer cells than Hex-B. Its antiproliferative action was comparable to that of the reference anticancer drug cisplatin [52].

Known alkaloids have been isolated from *A. uncinatus,* such as liriodenine, anonaine, norushinsunine, asimilobine and stepharine [47]. Anonaine (Figure 3) is also an aporphine alkaloid (benzylisoquinoline) with a significant antiplasmodial effect (IC_50_ = 23.2 μg/mL against *P. falciparum*) [53]. It can be found in several species of Magnoliaceae and Annonaceae, and has a large spectrum of bioactivities, including antiplasmodial, antibacterial, antifungal, and anticancer effects [54]. Alkaloids such as liriodenine, anonaine, and stepharine are relatively common in *Annona* species and contribute to the antiparasitic properties of the plants [55].

In the case of the rare butanolide, the alkaloid uncinine from *A. uncinatus* is interesting to underline because it has revealed marked cytotoxic properties, inhibiting the growth of HepG2 liver cancer cells (IC_50_ = 6.1 μg/mL) [46]. This original product combines a γ-alkylidene butenolide and pyrrolidinone fragments (Figure 3). Its total synthesis has been achieved [56], but its pharmacological properties are essentially unknown. This product should be studied further. In contrast, the alkaloid atherospermidine has been found in diverse plants, including *Artabotrys* species, such as *A. uncinatus* and *A. crassifolius* [57,58]. This compound can contribute to DNA damages in cells [59]. A related series of aporphine alkaloids also isolated from *A. uncinatus* has been named artacinatine [60]. The derivative 4,5-dioxoartacinatine has been isolated a few years later [61]. Artacinatine (Figure 2) can be found in other species, notably from the roots of *A. spinosus* together with artacinatine C [62], and from the stems and leaves of *A. hongkongensis* [63].

Lan and coworkers have identified more than 30 compounds from *A. uncinatus*, including (i) the alkaloid asimilobine which has antibacterial properties, (ii) the catecholic berberine alkaloid artavenustine, and (iii) a variety of classical sterol derivatives such as β-sitosterol and stigmasterol [61]. They also identified the unique derivative 24-methylenelanosta-7,9(11)-diene-3-one (Figure 3) analogous to the lanostane triterpene suberosol, which has antifungal and antiviral properties [64]. β-Sitosterol and related antimicrobial lipidic compounds have been identified from the leaves of *A. odoratissimus* (synonym) [65,66].

### 4.2. Terpenoids, Lignans and Flavonoids from Yingzhao

Lignans and flavonoids have been identified as well, including the dibenzylbutyrolactone lignans (iso)americanin, the flavonol glycoside artabotrysides A and B, and diverse flavonoids (taxifolin) and flavonoid glycosides such as quercetin/kaempferol/luetolin glycosides [44,67]. Artabotrysides A and B bear the same diglycoside moiety (3-rhamnosyl-(1→2)-alpha-L-arabinofuranoside) but a distinct flavonol core corresponding to quercetin for kaempferol, respectively (Figure 3). Among these compounds, taxifolin can be underlined as it is a prominent anti-inflammatory and antimicrobial compound [68]. Diverse antibacterial butyrolactone derivatives have been isolated also, such as tulipalin B and the compounds called artapetalins A-C with a unique β-methoxy-γ-methylene-substituted, α,β-unsaturated-γ-butyrolactone ring [69]. These compounds have been identified but not characterized from a pharmacological viewpoint. The semiterpenoid (*R*)-artabotriol has also been identified, together with artabotrycinol [70]. Artabotriol (Figure 2) is a precursor to the synthesis of tulipalin B and other bioactive natural products [71]. The two sesquiterpenoids artaboterpenoids A-B (Figure 2) have been isolated from the roots of the plants. The isomer (+)-artaboterpenoid B was shown to exhibit marked cytotoxicity toward several human tumor cell lines, with IC_50_ values in the range 1.38−8.19 μM [72]. This original bisabolene-derived sesquiterpenoid has not been reported in any other plant species. However, other sesquiterpenes have been identified from *A. hexapetalus,* including various yingzhaosu derivatives endowed with antiviral effects. This is the case of the derivative (8*S*,12*R*)-yingzhaosu C which revealed a noticeable antiviral effect against Coxsackie virus B3 (TC_50_ = 23.11 μM) [73].

Various volatile compounds have been identified from the flowers of the plant, including the abundant sesquiterpene called β-gurjunene (Figure 2) as a major component contributing to the antioxidant effect of the extract [74]. The related sesquiterpenoid globulol was also found, together with β-caryophyllene, well-known compounds with insecticidal activities (Figure 3). Sesquiterpene hydrocarbons and oxygenated sesquiterpene are commonly found in *Artabotrys* species [18,75]. They contribute largely to the antioxidant effects observed with essential oils from the plant [76]. Another interesting bioactive compound found in *A. uncinatus* is quebrachitol, a cyclic polyol (or cyclitol) which displays antidiabetic properties [77]. Other bioactive compounds found in yingzhao could be cited, such as the antibacterial butyrolactone tulipalin B [70], but in general, these compounds are trivial and are largely found in other species. In sharp contrast, there is a small group of unique products of major interest owing to their antimalarial properties: the yingzhaosu compounds detailed in the following section.

## 5. Yingzhaosu and Analogues

### 5.1. Discovery and Synthesis of Yingzhaosu A-D

The first two compounds in the series, yingzhaosu A and B (Figure 1), were isolated from the roots of the yingzhao plant in 1979 and described in two publications in Chinese [78,79]. The exact configuration of the compounds was not precisely known at that time. It was not clear if they corresponded to natural products or to artefacts formed in the root of *A. uncinatus* (synonym) during storage in the shade for two months, as mentioned later in a report [80]. They are effectively natural products from the plant. The two other compounds in the series, yingzhaosu C and D, were reported in 1989 by other Chinese chemists, 10 years after the discovery of parent and lead product yingzhaosu A [81,82]. Yingzhaosu C is a sesquiterpene peroxide, whereas yingzhaosu D is a sesquiterpenol (Figure 1). Since the discovery of the compounds, major efforts have been devoted to their total synthesis, but their pharmacological study has been largely neglected.

The first total synthesis of yingzhaosu A was presented in 1991, starting from the precursor *R*-(−)-carvone, a common monoterpene found in many plants [83]. *R*-(−)-carvone is known for its hypolipidemic, cytoprotective and sedative effects [84,85,86,87]. The total synthesis and X-ray diffraction analysis of synthetic yingzhaosu A provided key information about the stereochemistry of the product, indicating the S-configuration of the C-12 atom [83]. Subsequently, the synthesis of the diastereoisomeric yingzhaosu D was reported, starting from *S*-(−)-limonene, providing thus information about the configuration at positions C-4 and C-8 [88]. Then, the enantioselective synthesis of the four stereoisomers of yingzhaosu C was proposed [89], as well as epi-yingzhaosu C [90]. Over the years, significant efforts were deployed to optimize the total synthesis of these compounds and to propose synthetic analogues. The different synthetic routes have been optimized [91]. For example, a short and efficient synthesis of yingzhaosu C has been recently reported from the sesquiterpenoid obtained in one step [92].

**Figure 4:**
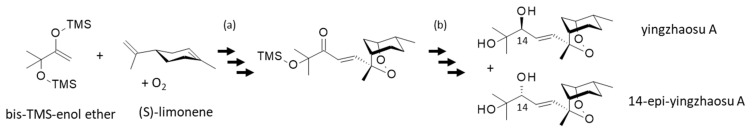
An efficient synthesis of yingzhaosu A from (S)-limonene and the TMS-protected enol ether in the presence of oxygen afforded the compound in 8 steps with an overall yield of 7.3% (**a**). The intermediate product (trimethylsilyloxy-enone peroxide) was converted into yingzhaosu A and its C14-epimer, via a chemoselective reduction [93] (**b**).

A remarkable effort led to the synthesis of yingzhaosu A in only 8 steps starting also from *S*-(−)-limonene, with an overall yield of 7.3% [93] (Figure 4). Limonene is particularly prone to addition of O_2_ and autoxidation [94,95]. Interestingly, this chemical work offered also the synthesis of the C(14)-epimer of yingzhaosu A, a compound characterized as a potent cytotoxic agent against KB nasopharyngeal cancer cells (ED_50_ = 36.6 and 0.57 μg/mL for yingzhaosu A and C(14)epi-yingzhaosu A, respectively). The epimer exhibited a higher antiplasmodial activity than the parent product (IC_50_ = 115 and 56 nM, against the chloroquine-resistant K1 strain of *P. falciparum*). The epimer was much more potent than the parent product in vivo against the chloroquine-sensitive *P. berghei* NY strain (ED_50_ = 250 and 90 mg/Kg for yingzhaosu A and C(14)epi-yingzhaosu A, respectively). However, the epimer remained much less active than the reference sodium artesunate in the same in vivo test (ED_50_ = 4.2 mg/Kg) [93]. This major work opened the door to the design of better analogues.

The synthesis of cyclic peroxide has attracted considerable interest in the chemistry field for different reasons (green chemistry, oxidation processes, …) [96,97]. The potent activity and medical value of the artemisinin and derivatives raise major interest as well. New artemisinin drug candidates are regularly proposed [98], and in the same vein, derivatives of yingzhaosu A and C, both containing a 1,2-dioxane unit, have been presented [99,100,101,102].

### 5.2. Synthesis and Pharmacology of Yingzhaosu Analogues

**Figure 5:**
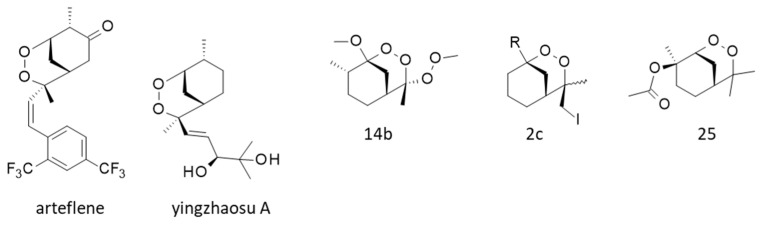
Synthetic derivatives of yingzhaosu A, such as arteflene (also known as Ro-42-1611) and the synthesized compounds 14b [103], 2c [25] and 25 [104].

Various cyclic peroxides structurally close to yingzhaosu A have been described, such as compound 14b (Figure 5), which was found to be active against *P. falciparum* (EC_50_ = 100 nM) but still less active than artemisinin (EC_50_ = 7.8 nM). Nevertheless, this compound presented a good cell selectivity, being weakly cytotoxic toward FM3A mouse mammary carcinoma cells (IC_50_ = 33 μM) [103]. Other potent compounds have been designed, in particular the cyclic peroxide 2c (Figure 5), which was found to remarkably inhibit the in vitro growth of *P. falciparum* (EC_50_ = 13 nM) with an efficacy comparable to that of artemisinin and a very high cellular selectivity [25,26]. Other related compounds active in vitro and in vivo were thus obtained, such as compound 25 (Figure 5) more potent than artemisinin against the parasite in vitro (EC_50_ = 3 and 10 nM, respectively) [104]. These chemical studies demonstrated that the endoperoxide scaffold of yingzhaosu A can be used as a template to design more potent analogues.

One particular compound derived from yingzhaosu A has been developed, the synthetic endoperoxide arteflene (Ro 42-1611) with a 1,2-dioxane unit (Figure 5). This compound is bioactivated in cells to generate a stable iron(II)-mediated carbon-centered radical [105]. Intracellular iron is believed to play a significant role in the bioactivation of certain endoperoxides, notably in the case of artemisinin [106]. This bioactivation process, occurring in hepatocytes, can lead to acute toxicity in the case of arteflene at high concentrations [107]. In the late 1990s, arteflene was considered a promising compound to treat malaria [108,109,110], and clinical trials were initiated [111,112]. However, the results were unconvincing, and the drug development stopped. Nevertheless, the arteflene program showed that yingzhaosu A can be used as a template to design innovative molecules [113].

## 6. Mechanism of Action of Yingzhaosu A

### 6.1. Reactivity in the Presence of Iron(II)

**Figure 6:**
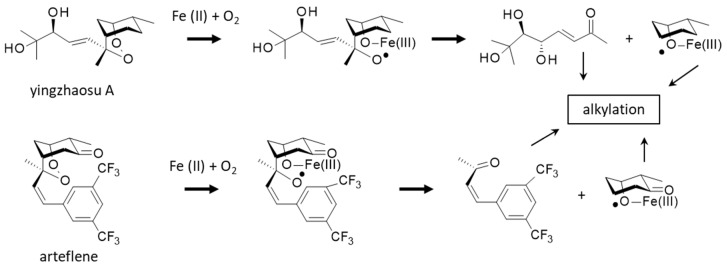
Reactions of yingzhaosu A or arteflene in the presence of iron and oxygen (Fenton reaction) lead to the formation of an oxygen-centered radical and then the release of alkylating species (unsaturated ketone and cyclohexyl radical), responsible for the parasiticidal properties (adapted from [114,115]).

A better knowledge of the molecular target and pathways involved in the antiplasmodial action of yingzhaosu A would help the design of analogues, but thus far, no study has been specifically devoted to elucidating the mechanism of action of the natural product. However, two important elements point toward the implication of iron and iron complexes in the bioactivity process. On the one hand, it is known that yingzhaosu A can be activated by an iron(II) compound in a Fenton-type reaction [114]. Yingzhaosu A is believed to undergo an iron(II)-induced degradation leading to the formation of two alkylating species, an unsaturated ketone and a cyclohexyl radical, as represented in Figure 6. Evidence for the generation of a cyclohexyl radical has been provided by the use of electron spin resonance (ESR). The same process can occur with arteflene in the presence of oxygen and iron chloride [114,115]. The reactive species thus generated would be responsible for the antiparasitic effects.

### 6.2. Reaction with Iron-Complexed Heme

**Figure 7:**
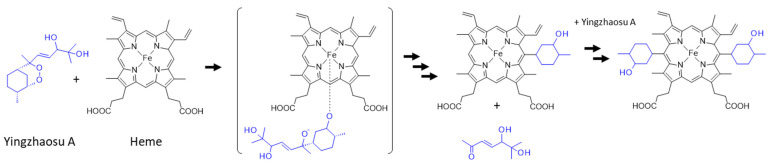
Proposed scheme for the reaction of yingzhaosu A with heme [116].

On the other hand, a recent study has pointed out the rearrangement and cleavage of yingzhaosu A in the presence of iron-bound heme. The authors built a heme-activatable probe based on the structure of yingzhaosu A to identify novel inhibitors of *P. falciparum*. They showed that the compound was attacked by heme to break the endoperoxide bond, thus generating sterically hindered tertiary oxygen-centered radicals. The yingzhaosu A molecule was broken into two parts after a rearrangement to remove the side chain, as represented in Figure 7 [116]. A similar endoperoxide reactivity-based FRET probe (with a bioinspired endoperoxide linker between donor and acceptor fluorophores) had been previously reported using an ozonide scaffold based on the architecture of artemisinin [117]. The yingzhaosu A-based probe is useful for identifying new bioactive compounds, but it is also informative on the reactivity of yingzhaosu A itself and its capacity to react with heme [116]. It will be interesting to determine whether the natural product can react similarly in cells. It is most likely that, as for artemisinin, the activation of yingzhaosu A requires the cleavage of the endoperoxide bridge in the presence of an iron source.

The role of the iron complex in the mechanism of action of endoperoxide drugs has been largely debated [118,119]. The bioreductive activation of endoperoxides of iron complexes, notably heme, leads to the generation of a radical species and then to alkylation of key proteins vital for the parasite. Moreover, upon alkylation, heme endoperoxide drugs can cause an imbalance in iron homeostasis, mitochondrial dysfunctions and toxic effects contributing to the cytocidal activity [120,121]. The heme molecule is considered a possible target (but probably not the sole target) of artemisinin-derived endoperoxides and analogous compounds [122]. Nevertheless, the role of heme is complex, and a recent study revealed that too much heme is not good for the antimalarial action of artemisinins [123]. The mechanism of action of artemisinin is pluri-factorial. It implicates a carbon radical and heme, but also interaction and interference with plasmodial proteins such as the sarcoplasmic endoplasmic calcium ATPase (SERCA), as well as an induced immunoregulation [124,125,126]. A similar complexity can be anticipated with yingzhaosu A.

## 7. Discussion and Conclusions

The medicinal plant *Artabotrys hexapetalus* (L.f.) Bhandari, or yingzhao in Chinese, has been known for decades and is extensively used in traditional medicine in Asia for the treatment of malaria and associated fevers. The plant is well known, but the various synonyms can result in confusion and complexity. The accepted name *A. hexapetalus* should be used primarily, not the synonyms, such as. *A. uncinatus* and *A. odoratissimus* in scientific communications. The plant has a large medicinal potential, well recognized in Asia, in particular for the treatment of parasitic diseases [127].

**Figure 8:**
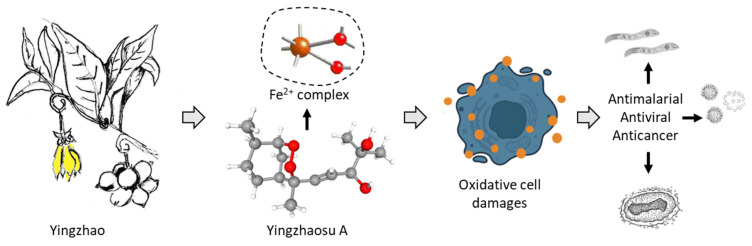
A schematic illustration of the pharmacological potential of yingzhaosu A, isolated from the plan yingzhao, for the treatment of parasitic diseases, viral diseases and cancer.

The chemical diversity of bioactive natural products identified from the plant is large. It is a rich source of secondary metabolites with diverse chemical classes (alkaloids, terpenoids, flavonoids, glycosides, …), as is frequently the case with medicinal species. The presence of many aporphine alkaloids is remarkable, notably in the oxazoloaporphine series (e.g., artabonatine). These products could be useful to combat various metabolic diseases, such as type 2 diabetes mellitus, endothelial dysfunction, hypertension and cardiovascular diseases [128]. There are also interesting flavones (artabotrysides A-B) and lactones (artapetalins A-C), not found or rarely in other plants, which would deserve further studies. However, with no doubt, the most interesting natural products isolated from the plant are the two endoperoxide-containing products yingzhaosu A and C and their derivatives yingzhaosu B and D. Immediately after their discovery, a major interest in these original compounds have sparkled amongst chemists towards the synthesis of these compounds and analogues. Important chemistry efforts have been devoted to optimizing total syntheses and to determine the exact stereoisomeric forms of the products. Analogues have been proposed in the early 2000s, leading to promising compounds in some cases [25,93,102]. However, the interest moved toward other endoperoxide-containing products analogous to artemisinin, probably because of the higher potency and clinical success of this exceptional molecule. The yingzhaosu compounds have been neglected over the past ten years. Their mechanism of action is not well defined, although it is likely as complex as that of artemisinin. It is time to breathe a new life into the yingzhaosu series with the design of new analogues and the application of modern technologies to better comprehend their mechanisms of action via network pharmacology studies and other multi-omic analyses (Figure 8).

The high potency of artemisinin and the recognized clinical efficacy of artemisinin combination therapy, which is recommended by the World Health Organization, contribute to promoting the design and development of novel compounds with a cyclic endoperoxide core. A huge diversity of compounds has been synthesized, including second and third generations of artemisinin derivatives (monomer, dimer, trimer), trioxolanes, tri- and tetra-oxanes, and a variety of non-artemisinin-derived synthetic endoperoxide-containing molecules [129,130,131,132,133]. Novel artemisinin derivatives are regularly proposed [134]. A large number of plant-derived endoperoxides, more than 200, have also been identified and studied, at least from the phytochemical aspect [135]. Among these efforts, there are opportunities to promote yingzhao and yingzhaosu compounds. The plant is readily available and even cultivated as an ornamental in the tropics. The products are affordable, with well-defined synthetic approaches. There is no reason not to promote the design of yingzhaosu A/C analogues. These endoperoxide compounds are important to combat parasitic diseases but also other diseases, such as respiratory diseases and cancers, as is the case for artemisinin and artesunate [9,136]. Hopefully, this review will contribute to restoring the prestige of yingzhaosu A and its analogues, whose compounds have been neglected for too long by the pharmacology community.

## Figures and Tables

**Table 1:** The plant yingzhao, accepted botanical names and synonyms.

Accepted Name	Synonyms *	Common Names **
*Artabotrys hexapetalus* (L.f.) Bhandari	*Annona hexapetala* L.f.	Tail grape (English); Hari champa, Kath champa, Madanmast (Hindi); Manoranidam (Tamil); Kothali-champa (Assamese); kenanga tanduk (Indonesian). Ylang-Ylang grimpant (French); Cay Móng rồng (Vietnamese); karawek (Thailand); iraniran noki, tsuru iraniran (Japanese); lanalana (Hawaiian); kathali champa, kaanthaali chaanpaa (Bengali).
	*Annona uncinata* Lam.	
	*Artabotrys hamatus* (Dunal) Blume	
	*Artabotrys intermedius* Hassk.	
	*Artabotrys odoratissimus* R.Br.	
	*Artabotrys odoratissimus* Wight & Arn.	
	*Artabotrys uncata* (Lour.) Baill.	
	*Artabotrys uncatus* (Lour.) Baill.	
	*Artabotrys uncinatus* (Lam.) Merr.	
	*Unona uncata* (Lour.) Dunal	
	*Unona uncinata* (Lam.) Dunal *Uvaria esculenta* Roxb. ex Rottler *Uvaria odoratissima* Roxb. *Uvaria uncata* Lour.	

* https://indiabiodiversity.org/species/show/228796 (accessed on 14 September 2022); http://www.theplantlist.org/tpl1.1/record/kew-2653287 (accessed on 14 September 2022). ** https://medplants.blogspot.com/2018/10/artabotrys-hexapetalus-champa-ylang.html (accessed on 14 September 2022).

**Table 2:** Pharmacological properties of *A. hexapetalus* extracts.

Pharmacological Activities	Comments	References
Antiparasitic	Extracts of the roots and fruits are used to treat malaria (*Plasmodium falciparum* infection) and leishmania (*Leishmania donovani infection).* Numerous antimalarial compounds characterized, principally the yingzhaosu.	[25,26,27,28]
Antibacterial	Activities against *Salmonella, Staphylococcus, Pseudomonas* and other bacteria reported with hydroalcoholic extracts of the flowers and leaves. Bactericidal effect of silver nanoparticles made with an aqueous extract of *A. hexapetalus*.	[29,30]
Antifungal	Activities against *Candida albicans*, *Aspergillus niger* and other fungi reported with a methanolic extract.	[21,24]
Antifertility	Reduction of sperm count and fertility with a hydroalcoholic leave extract, and modulation of the oestrus cycle. Regulation of steroidal hormone levels.	[31,32,33,34,35]
Anticancer	A fruit extract of *A. odoratissimus* reduced proliferation of MIA PaCa-2 pancreatic cancer cells and induced their apoptotic cell death.	[36]
Anti-ulcer and hepatoprotection	Protection against liver injuries and oxidative stress with a hydroalcoholic extract. Anti-inflammatory activity of an ethanolic extract of aerial parts. Cytoprotection conferred by antioxidant flavonoids and alkaloids.	[37,38,39,40]
Mosquito repellent	Essential oils made from leaves and stem bark extracts obtained by hydro-distillation have revealed mosquito repellency activity, attributed to the presence of β-caryophyllene oxide.	[16]

## Data Availability

Not applicable.
